# Knowledge, attitudes, and practices regarding antibiotic use among patients and their caregivers in India: a multicenter cross-sectional study

**DOI:** 10.1017/ash.2026.10822

**Published:** 2026-09-23

**Authors:** Krishna Suresh, Rajalakshmi Ananthanarayanan, Vettakkara Kandy Muhammed Niyas, Divyashree Shanthamurthy, Parvinder Kaur Chawla, Patricia Tayade, Adarsh A. K, Prasannakumar Palanikumar, Shobha Rana, Mary John, Namshid C. T, Selvakumar John Udaya Kumar

**Affiliations:** 1 Infectious Diseases, Trivandrum, India; 2 Kerala Institute of Medical Sciences: KIMSHEALTH Trivandrum, India; 3 MGM New Bombay Hospital, Vashi, India; 4 Fortis Hospital Mohali, India; 5 Christian Medical College and Hospital Ludhiana, India; 6 Aster MIMS: Malabar Institute of Medical Sciences Ltd, India; 7 Christian Medical College and Hospital: Christian Medical College Vellore, India

## Abstract

This multicenter study assessed knowledge, attitudes, and practices (KAP) regarding antibiotic use among 1,081 patients and caregivers. Despite positive attitudes, knowledge gaps were substantial. Education level significantly influenced KAP scores. Targeted awareness interventions are needed to improve knowledge gaps and to promote rational antibiotic use.

## Introduction

Antimicrobial resistance (AMR) is a growing global public health threat, with 1.27 million deaths directly attributable to drug-resistant infections and an additional 4.95 million associated with AMR in 2019.^1^ The problem is particularly severe in South Asian countries, including India, where antibiotic consumption rates are among the highest in the world.^2^ India’ high burden of infectious diseases, coupled with unregulated access to antibiotics, makes it a high-risk region for the emergence and spread of resistant bacterial strains.^3^Public knowledge, attitude, and practice (KAP) surveys provide valuable insights into awareness levels, misconceptions, and behavioral patterns regarding antibiotic use. Studies from across the world have highlighted widespread public misconceptions; for example, a community-based survey in coastal Karnataka, India, found that nearly half of respondents incorrectly believed antibiotics were effective against viruses, and a national survey in Bangladesh similarly reported limited public awareness of antibiotic resistance and its drivers.^4–9^

Existing research on antibiotic use has primary focused in general community populations. Hospital patients and caregivers are an important yet understudied group, despite their frequent interactions with healthcare providers and exposure to antibiotic treatments. Assessing their KAP regarding antibiotic use is essential. To our knowledge, this is among the first studies to examine antibiotic-use KAP across multiple states and hospital types in India

This study aims to evaluate the level of KAP regarding antibiotic use among hospital outpatients, inpatients, and their caregivers in multiple centers in India, identifying misconceptions, behavioral trends, and sociodemographic influences. The findings will help inform public health policies, awareness campaigns, and hospital-based interventions to promote rational antibiotic use and mitigate AMR.

## Methodology

This multicenter cross-sectional study was conducted across six hospitals in four states in India from November 2025 to February 2026. All six participating hospitals are located in urban centers, three are medical college affiliated teaching hospitals and three are private multi-/super-specialty hospitals without an attached undergraduate medical college. Adults (≥18 yr) attending outpatient or inpatient departments as patients or caregivers were included using convenience sampling during the study period. Healthcare professionals and patients considered acutely ill, including those admitted to critical care units, high-dependency units, or receiving end-of-life care were excluded from the study.

The sample size was calculated using a single population proportion formula assuming 50% prevalence of correct knowledge regarding antibiotic use, 95% confidence interval, and 5% allowable error. After adjusting for clustering and non-response, minimum sample size was estimated at 1,060 participants.

Data were collected through face-to-face interviews conducted by trained study coordinators using a structured, expert-validated questionnaire comprising 15 items across Knowledge, Attitude, and Practice domains (5 items each). The questionnaire was adapted from previously validated studies,^4–7^ and the validity of the content was assessed by experts in Infectious Diseases.

Descriptive statistics were used to summarize participant characteristics and KAP responses as frequencies and percentages. KAP scores were calculated by assigning 1 point for each correct/positive response and 0 for incorrect/negative responses. Scores were converted into percentages and categorized using Modified Bloom’ cut-off criteria as high (80%–100%), moderate (41%–79%), or low (0%–40%). Domain-specific scores were analyzed independently to identify specific gaps, while the overall KAP score was calculated as a composite of all 15 items to assess the general level of antibiotic awareness. The Kruskal–Wallis test with Dwass–Steel–Critchlow–Fligner posthoc analysis was used for group comparisons, with *P* < .05 considered statistically significant.

Ethics Committee approval was obtained from all six participating centers.

## Results

A total of 1,081 participants were included, of whom 53.6% were female, and the largest age group was 31–45 years (31.5%), followed by 46–60 years (29.1%), 18–30 years (25.0%), 61–75 years (12.5%), and over 75 years (1.9%). Knowledge regarding antibiotic use was limited. Only 33.8% correctly identified the purpose of antibiotics, 25.7% recognized that antibiotics are ineffective against viral infections, and although 53.7% were familiar with the term “antibiotic resistance,” only 21.4% correctly identified its contributing factors. Overall, 66.97.0% of participants had low knowledge scores, while only 16.09% achieved high scores.

Attitudes toward antibiotic use were generally positive, with 78.53% demonstrating positive attitudes. Most participants recognized the importance of consulting a doctor before taking antibiotics (92.2%) and completing the prescribed course (81.5%). Additionally, 80.6% considered non-prescription antibiotic availability a problem in India, 82.6% opposed pressuring physicians to prescribe antibiotics, and 74.4% were willing to participate in public awareness programs.

Self-reported practices revealed a mixed pattern. Although 77.9% reported completing prescribed antibiotic courses, inappropriate behaviors remained common, including over-the-counter antibiotic use without prescription (31.5%), reuse of leftover antibiotics (29.6%), sharing antibiotics (20.4%), and taking antibiotics for fever, cold, or sore throat without knowing whether they were necessary (38.4%). Overall, 61% demonstrated good practices.

**Figure 1.**
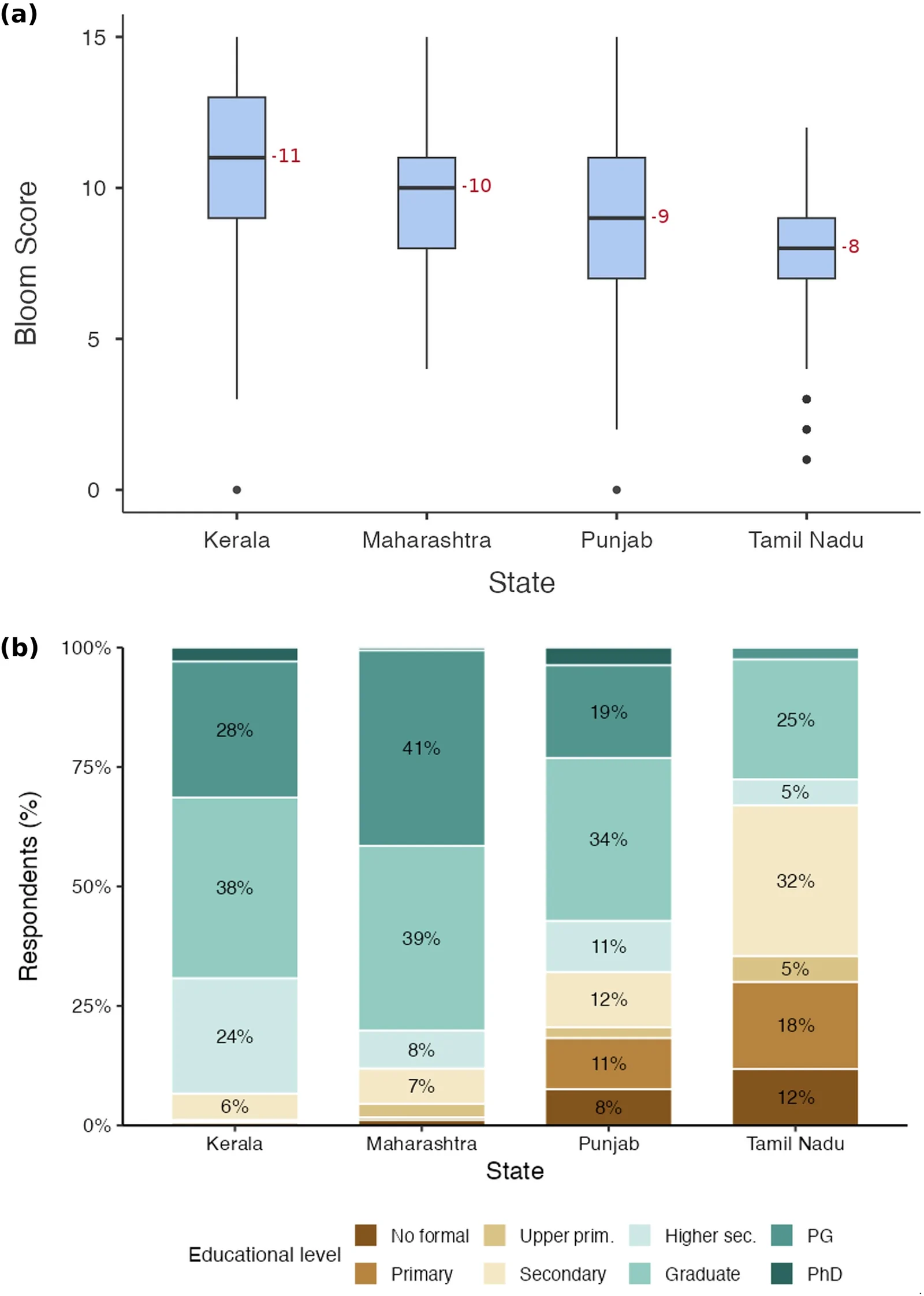
Figure 1 long description. Distribution of overall Modified Bloom’ KAP scores and educational attainment across participating states. (a) Box plots showing the distribution of Modified Bloom’ scores among respondents from participating states. Median KAP scores are indicated in red. (b) Distribution of respondents according to educational level across the participating states. Significant regional variation in KAP scores was observed (Kruskal–Wallis test, *P* < .001).

The overall KAP assessment showed that most participants had moderate scores (67.2%), followed by high (24.4%) and low (8.4%) scores. Female participants had slightly higher median Modified Bloom’ scores than males (10 vs 9; *P* = .005). Scores declined modestly with increasing age (Kruskal–Wallis χ^2^ = 33.3, df = 4, *P* < .001), from a median of 10 in young adults (18–30) and middle-aged adults (31–45) to 9 in older (46–60) and elderly (61–75) respondents, and 8 in the very elderly (>75). Educational attainment showed a strong positive association with KAP scores, with the highest scores observed among postgraduate and PhD participants (*P* < .001) (Table 1). Significant regional variation in Modified Bloom’ scores was observed across participating states (*P* < .001), with posthoc analysis confirming significant pairwise differences, indicating disparities in antibiotic-related knowledge, attitudes, and practices (KAP) across regions (Figure 1).

**Table 1.** Distribution of Modified Bloom’ score by highest level of education Table 1 long description.

Education level	n	Median	IQR
**No formal education**	55	7.0	5.0–8.0
**Primary (class 1–5)**	77	8.0	6.0–9.0
**Upper primary (class 6–8)**	25	9.0	7.0–9.0
**Secondary (class 9–10)**	137	9.0	7.0–10.0
**Higher secondary (class 11–12)**	147	10.0	7.0–12.0
**Under graduation**	372	10.0	8.0–12.0
**Post-graduation**	244	11.0	9.0–12.0
**PhD**	24	11.0	9.0–13.0
**Overall**	**1081**	**10.0**	**8.0–11.0**

## Discussion

This multicenter study identified substantial knowledge gaps regarding antibiotic use among hospital patients and caregivers in India. Despite largely positive attitudes, unsafe practices persisted. Only one-third of participants correctly understood the role of antibiotics, and misconceptions about viral infections were common, consistent with prior studies from South Asia.^4,5^ Although most participants recognized the importance of consulting physicians and completing prescribed antibiotic courses, self-medication, sharing antibiotics, and pressuring physicians for prescriptions revealed a disconnect between KAP. While overall KAP scores were predominantly moderate, domain-specific analysis showed that two-thirds of participants had poor knowledge scores, highlighting a critical deficit masked by relatively better attitude and practice scores.

Higher educational attainment was associated with better KAP scores, highlighting the need for targeted interventions among populations with lower literacy. Regional variations in KAP indicate disparities in healthcare access, public health messaging, and regulatory enforcement across states. This supports the need for context-specific strategies rather than uniform national interventions. This study has implications for antimicrobial stewardship programs, emphasizing the need to engage patients and caregivers. The findings also reinforce the role of hospitals as key settings for antimicrobial stewardship and patient education.

The study has some limitations. Self-reported practices may have been influenced by recall and social desirability bias, leading to underreporting of inappropriate antibiotic use. Additionally, economic characteristics were not assessed, limiting the evaluation of their influence on knowledge, attitudes, and practices regarding antibiotic use.

## Conclusion

Despite positive attitudes, significant unsafe practices, and knowledge gaps about antibiotic use persist among hospital patients and caregivers in India. Educational status significantly influences awareness. Targeted hospital- and community-based interventions are needed to promote rational antibiotic use and combat antimicrobial resistance.

## Supporting information

10.1017/ash.2026.10822.sm001 Suresh et al. supplementary material Suresh et al. supplementary material

## Supplementary material

The supplementary material for this article can be found at https://doi.org/10.1017/ash.2026.10822.

## Figure 1. Long description

The image contains two panels, each depicting different aspects of antimicrobial resistance awareness and educational attainment across four states in India. Panel A: A box plot showing the distribution of Modified Bloom’s KAP scores among respondents from Kerala, Maharashtra, Punjab, and Tamil Nadu. The y-axis represents the Bloom Score, and the x-axis lists the states. The median KAP scores for each state are indicated in red: Kerala has a median score of 11, Maharashtra 10, Punjab 9, and Tamil Nadu 8. The box plots show the interquartile range, with whiskers extending to the minimum and maximum values, and outliers marked as individual points. Panel B: A stacked bar chart displaying the distribution of respondents according to their educational level across the same four states. The y-axis represents the percentage of respondents, and the x-axis lists the states. The educational levels are color-coded: No formal education, Primary, Upper primary, Secondary, Higher secondary, Graduate, PG, and PhD. The percentages for each educational level vary by state, with Kerala having a higher proportion of respondents with higher secondary and graduate education, while Tamil Nadu shows a more even distribution across all educational levels.

Navigate back to Figure 1..

## Table 1. Long description

A table titled ‘Distribution of Modified Bloom score by highest level of education’ with five columns and eleven rows. The columns are labeled ‘Education level’, ‘n’, ‘Median’, and ‘IQR’. The rows list different education levels and their corresponding values. Row 1: No formal education, 55, 7.0, 5.0–8.0. Row 2: Primary (class 1–5), 77, 8.0, 6.0–9.0. Row 3: Upper primary (class 6–8), 25, 9.0, 7.0–9.0. Row 4: Secondary (class 9–10), 137, 9.0, 7.0–10.0. Row 5: Higher secondary (class 11–12), 147, 10.0, 7.0–12.0. Row 6: Under graduation, 372, 10.0, 8.0–12.0. Row 7: Post-graduation, 244, 11.0, 9.0–12.0. Row 8: PhD, 24, 11.0, 9.0–13.0. Row 9: Overall, 1081, 10.0, 8.0–11.0. The table shows the number of participants (n), the median score, and the interquartile range (IQR) for each education level. The data indicates that higher education levels are associated with higher median Modified Bloom scores.

Navigate back to Table 1..
