# Supplementary material for: Knowledge, attitudes, and practices regarding antibiotic use among patients and their caregivers in India: a multicenter cross-sectional study
Source: Antimicrob Steward Healthc Epidemiol. 2026 Sep 23;6(1):e292. doi: 10.1017/ash.2026.10822 (PMC13760294; doi:10.1017/ash.2026.10822)
Supplement: Supplementary file 1 [file S2732494X26108225sup001.docx]

A. Sociodemographic characteristics

1. Sex
a. Male
b. Female
c. Others
2. **Age (years)**

a. 18-30 (Young adults)
b. 31-45 (Middle-aged adults)
c. 46-60 (Older adults)
d. 61-75 (Elderly)
e. > 75 (Very elderly)

3. Highest level of education
a. No formal education
b. Primary
c. Upper primary
d. Secondary
e. Higher secondary
f. Under graduation
g. Post-graduation
h. PhD

4. State of residence __________________

**B. Knowledge regarding antibiotic use**

1. What are antibiotics used for?

a. bacterial infection

b. viral infection

c. both

d. don’t know

2. Can antibiotics cure viral infections such as the common cold or flu?

a. Yes

b. No

c. Don’t know

3. Are you aware of the term antibiotic resistance?

a. Yes

b. No

4. **If yes, do you think antibiotic resistance is a growing problem in India?**

a. Yes

b. No

c. Don’t know

5. If yes to Q3, which of the following do you think can cause antibiotic resistance?

a. Overuse of antibiotics

b. Incomplete course of antibiotics

c. Using left over antibiotics

d. All the above

e. Don’t know

**C. Attitude**

6. I would advise someone to complete their antibiotic course even if they feel better

a. Agree
b. Disagree

7. I believe it is important to consult a doctor before taking antibiotics.

a. Agree

b. Disagree

8. I put pressure on doctors to prescribe antibiotics

a. Agree
b. Disagree

9. I feel that the availability of antibiotics without a prescription is a problem in India

a. Agree

b. Disagree

10. I am willing to attend educational programs on the proper use of antibiotics

a. Agree

b. Disagree

**D. Practices regarding Antibiotic use**

11. Do you always complete the full course of antibiotics prescribed to you?

a. Yes

b. No

12. Have you ever used antibiotics left over from a previous prescription?

a. Yes

b. No

13. Have you ever shared your antibiotics with someone else?

a. Yes

b. No

14. Have you ever taken antibiotics to prevent an illness without a doctor’s prescription?

a. Yes

b. No

15. Have you ever taken antibiotics for conditions like fever, cold, or sore throat without knowing if it was necessary?

a. Yes

b. No
